# Stroke in India: A systematic review of the incidence, prevalence, and case fatality

**DOI:** 10.1177/17474930211027834

**Published:** 2021-07-02

**Authors:** Stephanie P Jones, Kamran Baqai, Andrew Clegg, Rachel Georgiou, Cath Harris, Emma-Joy Holland, Yogeshwar Kalkonde, Catherine E Lightbody, Pallab K Maulik, Padma MV Srivastava, Jeyaraj D Pandian, Patel Kulsum, PN Sylaja, Caroline L Watkins, Maree L Hackett

**Affiliations:** 1 6723 University of Central Lancashire, Preston, Lancashire, UK; 2 Society for Education, Action and Research in Community Health, Gadchiroli, India; 3 211065 The George Institute for Global Health, New Delhi, India; 4 University of New South Wales, Sydney, Australia; 5 Manipal University, Manipal, India; 6 All India Institute of Medical Sciences, New Delhi, India; 7 Christian Medical College, Punjab, India; 8 29354 Sree Chitra Tirunal Institute for Medical Sciences and Technology, Thiruvananthapuram, Kerala, India; 9 211065 The George Institute for Global Health, University of New South Wales, New South Wales, Australia

**Keywords:** Stroke, epidemiology, incidence, prevalence, case fatality, India, systematic review

## Abstract

**Background:**

The burden of stroke is increasing in India; stroke is now the fourth leading cause of death and the fifth leading cause of disability. Previous research suggests that the incidence of stroke in India ranges between 105 and 152/100,000 people per year. However, there is a paucity of available data and a lack of uniform methods across published studies.

**Aim:**

To identify high-quality prospective studies reporting the epidemiology of stroke in India.

**Summary of review:**

A search strategy was modified from the Cochrane Stroke Strategy and adapted for a range of bibliographic databases from January 1997 to August 2020. From 7717 identified records, nine studies were selected for inclusion; three population-based registries, a further three population-based registries also using community-based ascertainment and three community-based door-to-door surveys. Studies represented the four cities of Mumbai, Trivandrum, Ludhiana, Kolkata, the state of Punjab, and 12 villages of Baruipur in the state of West Bengal. The total population denominator was 22,479,509 and 11,654 (mean 1294 SD 1710) people were identified with incident stroke. Crude incidence of stroke ranged from 108 to 172/100,000 people per year, crude prevalence from 26 to 757/100,000 people per year, and one-month case fatality rates from 18% to 42%.

**Conclusions:**

Further high-quality evidence is needed across India to guide stroke policy and inform the development and organization of stroke services. Future researchers should consider the World Health Organization STEPwise approach to Surveillance framework, including longitudinal data collection, the inclusion of census population data, and a combination of hospital-registry and comprehensive community ascertainment strategies to ensure complete stroke identification.

## Introduction

Stroke is a significant global health problem and a major cause of mortality and morbidity in developed countries and increasingly in low-middle income countries (LMICs).^1^ Seventy percent of strokes occur in LMICs, and the subsequent disease burden is greater than that of high-income countries.^2^ Life expectancy in India has recently increased to over 60 years of age^3,4^ leading to an increase in age-related, non-communicable diseases including stroke;^5,6^ making stroke India’s fourth leading cause of death and fifth leading cause of disability.^7^

To address the rising burden of stroke in India, reliable data on stroke incidence, prevalence, and outcome is needed to inform healthcare policies and the organization of stroke services and to track the impact of any changes in care.^8^ In 2016, the Global Burden of Disease project^9^ estimated the number of incident cases of stroke in India to be 1,175,778. In a recent systematic review, consisting mainly of cross-sectional studies, the incidence of stroke in India was estimated to be between 105 and 152/100,000 people per year.^10^ However, there is a paucity of available data and a lack of uniform methods in published research.^11^ The aim of this systematic review was to identify high-quality prospective stroke epidemiology studies in India, to determine the crude and age-adjusted incidence and prevalence of stroke (providing sex disaggregated data where possible), and one-month case fatality.

## Methods

### Search strategy and study selection

The search strategy used terms for stroke that were taken from the Cochrane Stroke Strategy together with an adapted filter to identify epidemiology studies and additional terms for India.^12–14^ We adapted the strategy to search Medline (OVID), Embase (OVID), IMSEAR via Global Index Medicus, Science Citation Index Expanded (SCI-EXPANDED), Social Sciences Citation Index, and Arts & Humanities Citation Index within ISI Web of Science from and including January 1997 to August 2020. We chose 1997 as a starting year for this review as the Stroke Unit Trialists’ Collaboration systematic review^15^ was published in this year, providing a global standard for post-stroke care; recognizing that stroke was not only preventable, but treatable, a medical emergency, and that patients needed to be treated by stroke specialists or those with stroke specialist knowledge, skills, and experience. Studies from 1997 onwards would also be set against the background of the roll-out of thrombolysis in India and thus important for the outcomes of the review.

### Inclusion criteria

We included studies using prospective, consecutive recruitment with a pre-specified sampling strategy; studies with complete community-based case ascertainment with multiple overlapping sources; or non-community-based case ascertainment including case series and case–control studies with prospective, consecutive recruitment, grouped by location of recruitment e.g., acute hospital-based registry, rehabilitation-based registry.

Studies were included if participants had a confirmed history of stroke as defined by the World Health Organization (WHO)^16^ or as defined according to clinical criteria (confirmed by imaging, where possible) including cerebral infarction, intracerebral hemorrhage, subarachnoid hemorrhage, or uncertain pathological subtypes. There were no restrictions on age, sex, or other characteristics (e.g. degree of impairment post-stroke or interventions received).

### Exclusion criteria

Studies of mixed populations (e.g. stroke and head injury) were excluded unless separate results for people with stroke could be isolated. We excluded studies if they used cross-sectional recruitment, convenience sampling, retrospective recruitment, or only qualitative assessment. Randomized controlled trials and case studies were also excluded.

References were imported into EndNote^17^ and duplicates removed using the automated function in EndNote and then manually by an Information Specialist (CH). Forward and backward citation tracking was undertaken, and contact was made with experts.

### Data collection and analysis

**Figure 1.**
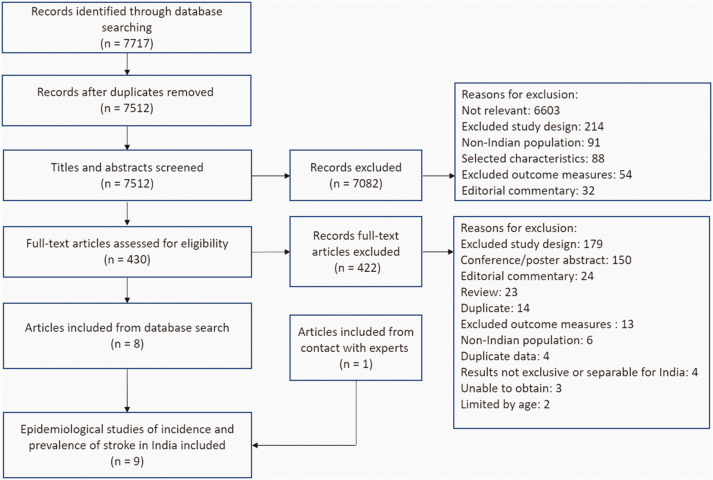
PRISMA flow diagram of study selection.

One reviewer (KB) screened all citations based on the title or abstract. Two independent reviewers (EJH, KP) screened a random selection of 20% of citations and inter-rater reliability calculated (ranging from 93% to 98%). All full text articles were read by KB and 10% were read by EJH and KP. Any disagreements were discussed with a third reviewer (SJ or MH) to reach consensus. Articles with evidence of overlapping recruitment sites, study dates, grant funding numbers, and similar or identical reported patient characteristics were considered to be from the same cohort, if not explicitly stated in the publications. The selection process is further described in Figure 1.

### Data extraction, selection, and coding

A bespoke data extraction form was pilot-tested by three reviewers (KB, EJH, SJ). Information from each study including author, year, study name, sample characteristics, and epidemiological data was extracted by KB and checked by EJH.

The protocol for the review was registered on PROSPERO.^18^ This systematic review has been reported following MOOSE guidelines for systematic reviews of observational studies^19^ and the Preferred Reporting Items for Systematic Review and Meta-Analyses (PRISMA) guidelines.^20^

### Assessment of risk of bias in included studies

The quality of the included studies methods was assessed using the Newcastle Ottawa Scale^21^ (Supplementary Table 1).

### Analysis

A priori we intended to perform a meta-analysis, but due to the small number of included studies, heterogeneity between study settings and designs, the included studies have been described narratively. The results are reported as presented in the original studies, with additional secondary analyses undertaken to calculate age-adjusted incidence to the WHO world standard population, crude prevalence (total number of stroke cases divided by total sample size per 100,000 population), case fatality, and associated 95% confidence intervals, where data were available.

## Results

We identified 7717 articles. Following screening, eight studies^22–29^ met the inclusion criteria (see Figure 1). An additional unpublished article, in press at the time of writing, was also identified through co-author JP.^30^

**Figure 2.**
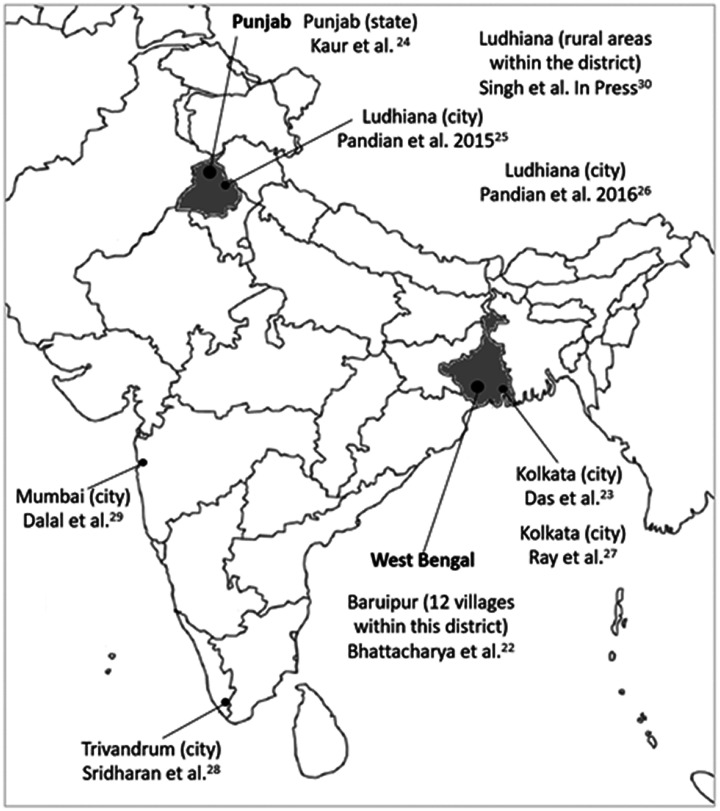
Location of studies assessing the incidence, prevalence, and outcome of stroke in India.

Study characteristics can be found in Table 1, and the location of the studies in India is shown in Figure 2. Three studies used population-based registries,^24,25,26^ a further three population-based registries also used community-based ascertainment^28,29,30^ and three conducted community-based door-to-door surveys.^22,23,27^ The population denominator (total sample size included in population-based registries or who agreed to participate in the door-to-door community studies) was 22,479,509 (mean 2,497,723 standard deviation (SD) 6,188,548). In the seven studies reporting this information, or available from respective population registries, the total number of females was 10,196,707 (48%). No studies reported the mean age of the population being studied.

**Table 1.** A summary of the characteristics of studies assessing the incidence, prevalence and outcome of stroke in India

Study and publication year	Study period	Location	Population type	Study design	Duration of surveillance (months)	Frequency of surveillance	Population denominator	Female *N* (%)
Bhattacharya et al.^22^	May 1992–April 1998	Baruipur	Rural	Community-based	72	Annual	20,717	9745 (47)
Das et al.^23^	March 2003–February 2005	Kolkata	Urban	Community-based	24	Twice a year	52,377	24,751 (47)
Dalal et al.^29^	January 2005–December 2006	Mumbai	Urban	Population-based registry	24	Continuous	156,861	77,031 (49)
Sridharan et al.^28^	January 2005–February 2005	Trivandrum	Urban and rural	Population-based registry	6	Continuous	925,867	470,096 (51)
Ray et al.^27^	March 2003–February 2010	Kolkata	Urban	Community-based	84	Biannually	100,802	47,593 (47)
Pandian et al.^25^	March 2010–March 2011	Ludhiana	Urban	Population-based stroke registry	12	Continuous	935,925	404,051 (43)
Pandian et al.^26^	March 2011–March 2013	Ludhiana	Urban	Population-based stroke registry	24	Continuous	1,065,127	NS
Kaur et al.^24^	March 2010–March 2013	Punjab	Urban and Rural	Population-based registry	24	Continuous	18,962,055	9,163,440 (48)
Singh et al.^30^	December 2016– November 2018	Ludhiana	Rural	Population- based registry	28	Continuous	259,778	NS

NS: not stated.

The total number of people identified with incident stroke was 11,654 (mean 1294 SD 1710). In the six studies reporting age data, the mean age of those with stroke was 62.2 years, and in the eight studies reporting sex data, the total number of females was 3344 (41%) (Table 2). Stroke was classified by a neurologist or physician based on CT and/or MRI scans in five studies,^23,24,26,27,30^ the remaining used a range of case ascertainment methods described in Supplementary Table 1. The proportion of people undergoing CT or MRI ranged from 38%^27^ to 95%.^26^ Rates of ischemic stroke ranged from 65% in Kolkata^23^ to 84% in Trivandrum,^26^ and intracerebral hemorrhage from 11% in Trivandrum^26^ to 35% in Kolkata.^23^ Only three studies^22,23,29^ reported hospitalization rates ranging from 26%^22^ to 69%.^23^

**Table 2.** A summary of the epidemiological data from studies assessing the incidence, prevalence, and outcome of stroke in India

Study and publication year	No. of cases of stroke identified	Imaging not available *N* (%)	Stroke type *N* (%)	Mean age (years) *N* (SD)	Female *N* (%)	Crude annual incidence/ 100,000 *N* (95% CI)	Sex-disaggregated incidence rate male/100,00 *N* (95% CI)	Sex-disaggregated incidence rate female/ 100,000 *N* (95% CI)	Age-adjusted incidence/ 100,000 *N* (95% CI)	Crude prevalence/ 100,000 *N* (95% CI)	Age-adjusted prevalence/ 100,000 *N* (95% CI)	One-month case fatality % (95% CI)
Bhattacharya et al.^22^	128	NS	NS	61 (NS)	60 (47)	124 (NS)	124 (NS)	123 (NS)	108 (88–130)^a^	618 (509–1707)^a^	NS	18 (NS)
Das et al.^23^	247	81 (33)	IS 108 (65) ICH 58 (35)	NS	110 (45)	123 (103–233)	100 (75–130)	149 (117–166)	145 (120–175)	472 (41–534)	545 (479–617)	41 (31–54)
Dalal et al.^29^	456	0 (0)	IS 366 (80) ICH 81 (18) US 9 (2)	66 (14)	218 (48)	145 (120–170)	149 (120–170)	141 (120–160)	92 (74–113)^a^	291 (264–318)^a^	NS	30 (NS)
Sridharan et al.^28^	541	169 (31)	IS 311 (84) ICH 43 (12) SAH 18 (4)	67 (NS)	279 (52)	117 (NS)	115 (NS)	119 (NS)	135 (123–146)	58 (53–63)^a^	135 (123–146)	27 (NS)
Ray et al.^27^	763	NS	NS	NS	341 (45)	108 (88–131)	113 (86–146)	102 (75–136)	141 (114–171)	757 (702–817)^a^	NS	42 (38–46)^a^
Pandian et al.^25^	493	NS	NS	58 (15)	185 (38)	NS	NS	NS	NS	53 (48–57)^a^	NS	NS
Pandian et al.^26^	3441	2122 (62)	IS 976 (74) ICH 290 (22) SAH 53 (4)	NS	NS	140 (133–147)	151 (141–161)	106 (97–115)	130 (123–137)	323 (312–334)^a^	NS	22 (21–23)^a^
Kaur et al.^24^	4989	26 (0)	IS 3260 (66) ICH 1656 (33) CVT 47 (1)	59 (15)	1865 (37)	NS	NS	NS	NS	26 (25–27)^a^	NS	NS
Singh et al.^30^	596	NS	NS	62 (15)	286 (48)	172 (NS)	170 (NS)	173 (NS)	209 (NS)	229 (211–248)^a^	NS	NS

NS: not stated; IS: ischemic stroke; ICH: intracerebral hemorrhage; CVT: cerebral venous thrombosis; NA: no scan available; US: unspecified stroke type; SD: standard deviation; CI: confidence interval.

a Calculated manually based on information in article.

In seven studies, the crude annual incidence rate ranged from 108/100,000^27^ to 172/100,000^30^ people per year. Age-adjusted incidence was reported in the same seven studies and ranged from 92/100,000 in the city of Mumbai^29^ to 209/100,000 in two rural blocks in Ludhiana District.^30^

Only one study reported the crude prevalence rate.^23^ We calculated crude prevalence rates for all the other studies, and this ranged from 26/100,000^24^ to 757/100,000^27^ people per year.

Across most studies, stroke incidence rates were higher for men (see Table 2). The exception was 100/100,000 for men and 149/100,000 for women in Kolkata;^23^ 115/100,000 and 119/100,000 in Trivandrum,^28^ and 170/100,000 and 173/100,000, respectively, in the rural villages of Ludhiana.^30^ One-month case fatality rates ranged from 18%^22^ to 42%^27^ and were highest in the studies based in Kolkata (41–42%),^23,27^ where premature stroke deaths were twice as high amongst men than women.

### Methodological quality

All studies were deemed “Good Quality”^21^ (Supplementary Table 1). The three community studies used door-to-door screening questionnaires; one study^22^ used the WHO proforma (1981),^31^ but the other two^23,27^ did not provide details on the screening questionnaire used. All studies (excluding one^23^) followed all three steps of the WHO STEPwise approach to Surveillance (STEPS) framework.^32^ Most studies used the WHO world standard population to calculate incidence.^23,26,27,28,30^ Age-adjusted incidence was recalculated for two studies that originally used Segi’s 1996 world population^29^ and the USA population 1990.^22^

## Discussion

We found limited epidemiological data, representing only the four cities of Mumbai, Trivandrum, Ludhiana, Kolkata, the state of Punjab, and 12 villages of Baruipur in the state of West Bengal, leaving the vast majority of India without high-quality epidemiological stroke data. Crude incidence rates ranged from 108^27^ to 172/100,000^30^ and age-adjusted incidence rates between 92^29^ and 209/100,000,^30^ similar to those previously reported.^10^ There were large variations in the crude prevalence for stroke from 26^24^ to 757/100,000,^27^ similar but larger than that reported in a previous systematic review (44–559/100,000).^10^ Overall, sex disaggregated incidence rates were only slightly higher for males (100–170/100,000 than females 102–173/100,000^23,27,30^) and one-month case fatality varied from 18% to 42% higher than that observed in developed nations^33,34^ and was twice as high in men than women^22,27^ in comparison to higher premature case fatality rates in women, globally.^35^ Higher one-month case fatality rates, particularly amongst men, requires further research to improve access to high-quality specialist stroke care and secondary prevention measures and necessitates the collection of high-quality epidemiological data ensuring that all deaths are accurately coded for all members of the population. Given the heterogeneity of the available data from only four of the 28 States and 8 Union Territories of India, there is insufficient high-quality evidence to guide stroke policy, service planning, and delivery and its evaluation in India.

The three door-to-door community studies took place in the state of West Bengal and reported greater crude prevalence rates (472, 618, and 757) than in the population-based stroke registry studies. The Million Death Study^36^ found that a third of premature stroke deaths in India occurred in North-Eastern states, including West Bengal. Reasons for this may include ethnic differences in North-Eastern states of India, where the population has greater rates of hypertension^37,38^ and dietary factors such as higher salt intakes.^35^ Whilst this region has higher reported stroke incidence, prevalence, and premature mortality rates,^22,23,27,33^ it is difficult to make comparisons with other areas due to a lack of data and differences in study designs.

The population-based registry studies in this review identified very small numbers of people who had a stroke and did not attend hospital.^24–26^ However, reports from India suggest that many people who experience a stroke do not access hospital services for multiple reasons including limited awareness of stroke symptoms or stroke being an emergency, large distances to travel between home and hospital, a lack of ambulance staff and transportation, the availability of alternative [non-hospital] therapies that people may consider effective after stroke, and limited finances to cover the cost of care.^28,38,39^ There were also limited data on the rates of hospitalization.

While the population-based stroke registry studies in Ludhiana and Punjab used newspaper advertisements every six months to identify people with stroke who sought treatment elsewhere, only “a few” to 15 people^24–26^ were identified using this method. Two of the included studies had notably lower percentages of females with stroke compared to females in the surveillance population observed.^24,25^ This is congruent with reports suggesting that women in India can experience discrimination in accessing healthcare.^40^ The mean age of stroke survivors (62.2 years) was younger than the global mean age of people with stroke.^41^ In India, nearly one-fifth of patients with first ever strokes admitted to hospital are under 40 years of age, and this often has a devastating impact on the future health, finances, and welfare of individuals and their families.^42^

Limitations of this review include being unable to obtain full copies of three potential articles for inclusion (Supplementary Table 2) despite contacting national libraries and authors for further information. Whilst we made contact with experts, it is possible that we have not identified all relevant articles published in non-indexed journals.

Given what we know about hospital stroke presentations in India, even the data from the studies included in this review are likely conservative estimates of the true stroke incidence, prevalence, and outcome. When identifying the burden of stroke across other States and Union Territories of India, special attention will need to be paid to additional recruitment methods to those outlined in the WHO STEPS framework^31^ to identify all of those with stroke not receiving hospital care. The population-based registry studies included in this review used various forms of community-based ascertainment strategies including contact with healthcare facilities (imaging centers, rehabilitation facilities, and general physicians),^28,29^ the use of Accredited Social Health Activists,^30^ and verbal autopsies (particularly in rural or remote areas where reliable stroke data and death registers were not available).^28^ To improve stroke services and to generate reliable data, future researchers should consider using multiple methods to ensure complete case ascertainment. An example of this is the National Population Based Stroke Registry,^43^ which aims to expand epidemiological, clinical, and public health research on stroke.

## Supplemental Material

sj-pdf-1-wso-10.1177_17474930211027834 - Supplemental material for Stroke in India: A systematic review of the incidence, prevalence, and case fatality Click here for additional data file. Supplemental material, sj-pdf-1-wso-10.1177_17474930211027834 for Stroke in India: A systematic review of the incidence, prevalence, and case fatality by Stephanie P Jones, Kamran Baqai, Andrew Clegg, Rachel Georgiou, Harris Cath, Emma-Joy Holland, Yogeshwar Kalkonde, Catherine E Lightbody, Pallab BK Maulik, Padma MV Srivastava, Pandian J Durai, Patel Kulsum, PN Sylaja, Caroline L Watkins, Hackett Maree L and on behalf of the NIHR Global Health Research Group on IMPROVIng Stroke carE in India (IMPROVISE) Collaboration in International Journal of Stroke
