# Supplementary material for: Stroke in India: A systematic review of the incidence, prevalence, and case fatality
Source: Int J Stroke. 2021 Jul 2;17(2):132–40. doi: 10.1177/17474930211027834 (PMC8821978; doi:10.1177/17474930211027834)
Supplement: sj-pdf-1-wso-10.1177_17474930211027834 - Supplemental material for Stroke in India: A systematic review of the incidence, prevalence, and case fatality [file sj-pdf-1-wso-10.1177_17474930211027834.pdf]

Supplementary Table 1. A summary of the methodological quality of included studies according to the Newcastle Ottawa scale for cohort studies.

**Study**<br>**Author Year**	**Selection**<br>**Representativeness of the exposed cohort**	**Selection**<br>**Selection of the non-exposed cohort**	**Selection**<br>**Ascertainment of exposure**	**Selection**<br>**Demonstration that outcome of interest was not present at start of study**	**Comparability**<br>**Comparability of cohorts on the basis of the design or analysis**	**Outcome**<br>**Assessment of outcome**	**Outcome**<br>**Was follow-up long enough for outcomes to occur**	**Outcome**<br>**Adequacy of follow-up of cohorts**
Bhattacharya et al. 2005	B) Somewhat representative of the average population of West Bengal (not all the social classes may have been represented due to cluster sampling)	A) Drawn from the same community	B) Structured interview (door-to-door survey conducted by trained doctors in neurology, using the WHO proforma (1981))	A) Yes	A) Study reports age and sex<br>B) Study reports stroke risk factors	B) Record linkage<br>(all villagers suspected to be suffering from stroke via the questionnaires were examined clinically by trained doctors in neurology)	A) Yes (follow-up data were obtained from every available stroke patient on the 30th day and after one year from the enrolment of stroke cases)	D) No statement (1% of the population did not participate, but there were no reported dropouts in the 99% that did)
Das et al. 2007	A) Truly representative of Kolkata	A) Drawn from the same community	B) General screening and structured interview (door-to-door survey with the help of a general screening questionnaire by four field workers headed by a neurologist)	A) Yes	A) Study reports age and sex	B) Record linkage<br>(door-to-door survey used with general screening questionnaire. Neurologists clinically examined positive cases and recorded clinical details)	A) Yes (2 years)	D) No Statement (2% of households did not participate, but there were no reports of dropouts in the 98% that did)

Dalal et al. 2008	B) Somewhat representative of the population of Mumbai (the study was conducted in one ward (H-ward) of Mumbai. May not be representative of socioeconomic status).	A) Drawn from the same community	A) Secure records (hospital scan reports by medical practitioners and death certificates)	A) Yes	A) Study reports age and sex	A) Independent assessment	A) Yes (2 years)	A) Complete follow-up - all subjects accounted for (due to use of population-based registry)
Sridharan et al. 2009	A) Truly representative of Trivandrum	A) Drawn from the same community	A) Secure records (hospital scan reports by neurologist and death certificates)	A) Yes	A) Study reports urban and rural locations, age and sex<br>B) Study reports types of stroke and risk factors	A) Independent blind assessment	A) Yes (6 months)	A) Complete follow-up - all subjects accounted for (due to use of population-based registry)
Ray et al. 2013	A) Truly representative of Kolkata	A) Drawn from the same community	B) Structured interview (standard questionnaire used by one doctor and 4 nonprofessional workers)	A) Yes	A) Study reports age and sex	B) Record linkage (door-to-door survey used with a standard questionnaire. Used by a team of one doctor and four nonprofessional workers)	A) Yes<br>(7 years)	B) Subjects lost to follow-up unlikely to introduce bias - small number lost 89 (10%)

Pandian et al. 2015	B) Somewhat representative of the average population of Ludhiana (females and stroke patients unable to attend hospital may be underrepresented)	A) Drawn from the same community	A) Secure records (hospital scan reports by radiologist and death certificates)	A) Yes	A) Study reports age and sex<br>B) Study reports religion, education, occupation, and hospital type admitted to	A) Independent assessment	A) Yes (8 months)	A) Complete follow-up - all subjects accounted for (due to use of population-based registry)
Pandian et al. 2016	B) Somewhat representative of the average population of Ludhiana (Females and stroke patients unable to attend hospital may be underrepresented)	A) Drawn from the same community	A) Secure records (hospital scan reports by radiologist and death certificates)	A) Yes	A) Study reports age and sex<br>B) Study reports spatial distribution of patients	A) Independent blind assessment	A) Yes<br>(31 months)	A) Complete follow-up - all subjects accounted for (due to use of population-based registry)
Kaur et al. 2017	B) Somewhat representative of the average population of Punjab (females and stroke patients unable to attend hospital may be underrepresented)	A) Drawn from the same community	A) Secure records (hospital scan reports by radiologists)	A) Yes	A) Study reports urban and rural locations<br>B) Study reports demographic details, types of stroke and risk factors	B) Record linkage (door-to-door survey used with general screening questionnaire. Neurologists clinically examined positive cases and recorded clinical details)	A) Yes (24 months)	A) Complete follow-up - all subjects accounted for (due to use of population-based registry)

Singh et al. In press[30]	A) Truly representative of rural areas of Ludhiana	A) Drawn from the same community.	A) Secure record (hospital records, case record forms by neurologists and verbal autopsy)	A) Yes	A) Study reports age, sex and risk factors	A) Independent blind assessment	A) Yes (28 months)	A) Complete follow-up - all subjects accounted for (due to use of population-based registry)

Supplementary Table 2. Studies unable to obtain

Bhalla D, Marin B, Cabanac M.-D, Preux P.-M. Stroke profile in Afghanistan, Nepal, and India. *Revue Neurologique* 2012;168(2) https://doi.org/10.1016/j.neurol.2012.01.403.
Lewington S, Gajalakshmi V, Lacey B, Sherliker P, Vendhan K, Peto R. Blood Pressure, Body-Mass Index and Vascular Mortality in 331,000 Indian Non-Smokers: The Chennai Prospective Study. *Global Heart* 2016;11(2):e134.
Suresh D and John KR. Study to assess the prevalence of stroke in the Kattankulathur block of Kancheepuram district, Tamil Nadu, India. *International Journal of Pharma and Bio Sciences* 2016;7(4):805-809. doi.org/10.22376/ijpbs.2016.7.4.b805-809.
